# Evaluating AI Competence in Specialized Medicine: Comparative Analysis of ChatGPT and Neurologists in a Neurology Specialist Examination in Spain

**DOI:** 10.2196/56762

**Published:** 2024-11-14

**Authors:** Pablo Ros-Arlanzón, Angel Perez-Sempere

**Affiliations:** 1 Department of Neurology, Dr. Balmis General University Hospital, C/ Pintor Baeza, Nº 11, Alicante, 03010, Spain, 34 965933000; 2 Department of Neuroscience, Instituto de Investigación Sanitaria y Biomédica de Alicante, Alicante, Spain; 3 Department of Clinical Medicine, Miguel Hernández University, Alicante, Spain

**Keywords:** artificial intelligence, ChatGPT, clinical decision-making, medical education, medical knowledge assessment, OpenAI

## Abstract

**Background:**

With the rapid advancement of artificial intelligence (AI) in various fields, evaluating its application in specialized medical contexts becomes crucial. ChatGPT, a large language model developed by OpenAI, has shown potential in diverse applications, including medicine.

**Objective:**

This study aims to compare the performance of ChatGPT with that of attending neurologists in a real neurology specialist examination conducted in the Valencian Community, Spain, assessing the AI’s capabilities and limitations in medical knowledge.

**Methods:**

We conducted a comparative analysis using the 2022 neurology specialist examination results from 120 neurologists and responses generated by ChatGPT versions 3.5 and 4. The examination consisted of 80 multiple-choice questions, with a focus on clinical neurology and health legislation. Questions were classified according to Bloom’s Taxonomy. Statistical analysis of performance, including the κ coefficient for response consistency, was performed.

**Results:**

Human participants exhibited a median score of 5.91 (IQR: 4.93-6.76), with 32 neurologists failing to pass. ChatGPT-3.5 ranked 116th out of 122, answering 54.5% of questions correctly (score 3.94). ChatGPT-4 showed marked improvement, ranking 17th with 81.8% of correct answers (score 7.57), surpassing several human specialists. No significant variations were observed in the performance on lower-order questions versus higher-order questions. Additionally, ChatGPT-4 demonstrated increased interrater reliability, as reflected by a higher κ coefficient of 0.73, compared to ChatGPT-3.5’s coefficient of 0.69.

**Conclusions:**

This study underscores the evolving capabilities of AI in medical knowledge assessment, particularly in specialized fields. ChatGPT-4’s performance, outperforming the median score of human participants in a rigorous neurology examination, represents a significant milestone in AI development, suggesting its potential as an effective tool in specialized medical education and assessment.

## Introduction

Recent advancements in natural language processing, particularly the development of large language models (LLMs), have markedly transformed the capabilities of computational linguistics. Among these, ChatGPT, developed by OpenAI, stands out as a leading example, leveraging advanced deep learning techniques to emulate humanlike text generation. Introduced in late 2022, ChatGPT has quickly gained recognition for its ability to produce coherent and contextually relevant responses, owing to its training on a broad dataset [1]. This versatility has made ChatGPT a valuable tool in numerous fields, including medicine.

In the medical field, ChatGPT’s potential has been explored through its application in clinical settings and medical examinations, where it has demonstrated a notable proficiency in addressing complex medical and dental queries [2-9]. This has sparked interest in its role in improving medical education and training and support clinical decision-making.

In Spain, the process of obtaining a public position as a medical specialist in the public health service involves a competitive examination, which is administered independently across various regions. This is exemplified in the Valencian Community, where the selection of neurology specialists depends on an examination, encompassing both health legislation and clinical neurology questions. The examination is a critical component for securing a position in the public health care system, similar to a civil service examination, and is highly competitive. The candidates are already accredited neurologists with a minimum of 4 years of residency and at least 1 year of professional experience.

Despite numerous studies examining the performance of ChatGPT in various medical examinations, a significant gap remains in comparing its capabilities with the real performance and results of highly qualified and specialized clinicians in regional specialty examinations. This study specifically addresses this gap by comparing ChatGPT’s performance with that of practicing neurologists in the Valencian Community’s neurology specialist examination. The primary objective is to evaluate whether ChatGPT can match or surpass human expertise in this context. Additionally, we aim to assess the consistency and improvement in responses between ChatGPT versions 3.5 and 4. Our a priori hypotheses are as follows: (1) ChatGPT-4 will outperform ChatGPT-3.5, demonstrating improved accuracy and reliability, and (2) ChatGPT-4 will perform comparably to human neurologists. This analysis seeks to provide insights into the potential and limitations of artificial intelligence (AI) in specialized medical knowledge assessment and its implications for medical education and practice.

## Methods

### Study Design

We conducted a detailed comparative analysis to evaluate the performance of ChatGPT against board-certified neurologists in the 2022 Valencian Community neurology specialist examination [10]. This examination is a credentialing examination that grants a job position in the public health system as a neurology specialist within the Valencian Community, rather than a medical licensing examination. Candidates who sit for this examination are already certified neurologists, having completed a minimum of 4 years of residency and at least 1 year of professional experience. Therefore, this examination is more specialized and competitive compared to typical specialty board examinations that grant the initial permission to practice. The 2022 examination employed a multiple-choice format, with 77 out of the original 80 questions considered for scoring, as 3 were invalidated due to errors in question formulation. A total of 120 practicing neurologists took the examination, competing for only 38 available job positions. The results of the individual examinations of each participating neurologist are publicly available on the Department of Health’s website [11].

The Valencian Health Service is one of the 17 regional health services in Spain, providing universal health care to both residents and travelers in the Valencian Community. This region, located on the eastern Mediterranean coast of Spain, has a population of more than 5.2 million inhabitants and attracts around 28.5 million tourists annually. The scope and geographic reach of the Valencian Health Service include all health care facilities within this region, making the credentialing examination crucial for those seeking to work in these public health care institutions.

### Multiple-Choice Question Examination

The examination adopted a scoring system where the maximum attainable score was 10, achievable by correctly answering all questions. Unanswered questions were not penalized. The scoring system penalized wrong answers: for every 3 wrong answers, the score for 1 correct answer was subtracted. Score = (N_correct_ – 1/3 N_wrong_) × 10/N_total_, where “N” represents the numbers of correct (N_correct_) and wrong (N_wrong_) answers and the total number of questions (N_total_). The test began with 12 questions on general public and health legislation topics, followed by 65 questions focused on clinical neurology, assessing both theoretical knowledge and clinical reasoning. Participants with a score higher than 4.5 points passed the examination [10].

### Data Collection and Assessment

We compiled the scores of the 120 participating neurologists, which are publicly available (Table S1 in Multimedia Appendix 1). To assess the performance of GPT-3.5 and GPT-4, we used their respective application programming interfaces (APIs). Two independent researchers, PRA and APS, tasked the ChatGPT versions 3.5 and 4 with answering the examination’s multiple-choice questions. This study was conducted in December 2023 and used the LLM versions available at that time.

#### Prompt Engineering

For consistency, each version of ChatGPT was given the same set of prompts. The initial prompt provided a brief context of the examination question and instructed the AI to select the best answer (see Supplement 1 in Multimedia Appendix 1).

#### Interface Version

We utilized the paid subscription API for both ChatGPT-3.5 and ChatGPT-4, ensuring access to the most advanced features available. The settings used included the default temperature settings to maintain consistency and comparability between responses.

#### Language Settings

Both input and output languages were set to Spanish to match the language of the original examination. This ensured that the AI models processed and responded to the questions in the same language as the neurologists.

#### Trial Repetitions

Each ChatGPT version was tested twice independently to account for any variability in responses. This involved rerunning the entire set of examination questions with the same prompts. For each trial, the responses were recorded and analyzed separately to evaluate consistency and performance.

#### Efforts to Chain Prompts

No prompt chaining was employed in this study. Each question was presented individually, and the AI’s responses were based solely on the information provided in the individual prompts.

#### Details of Trials

In total, 4 sets of responses were generated (2 for each version of ChatGPT). Each trial was conducted independently by the researchers to avoid memory bias or influence from previous attempts. The answers were then compiled and compared against the correct answers to calculate the scores.

### Question Complexity Classification

Questions in the examination were categorized according to the principles of Bloom’s Taxonomy [12], a framework for learning and evaluation. This classification differentiated between questions testing lower-order thinking skills, such as recall and basic understanding, and those measuring higher-order thinking skills, such as application, analysis, and evaluation. The classification process involved the following steps. Two independent researchers, PRA and APS, assigned Bloom’s Taxonomy classifications to each examination question. To ensure consistency and accuracy in the classification, the initial assignments by both researchers were compared. Any discrepancies in classification were discussed in consensus meetings between the researchers until an agreement was reached. After resolving discrepancies, the final classifications were used in the analysis. These classifications were then used to evaluate the performance of ChatGPT-3.5 and ChatGPT-4 across different levels of cognitive tasks.

### Statistical Analysis

The statistical analysis of the data was conducted using R software, version 4.2.1 (R Foundation for Statistical Computing) [13].

We checked the data’s normality using the Kolmogorov-Smirnov test. To assess the consistency of responses within each ChatGPT version across different trials, we calculated the κ coefficient for each model. Specifically, we compared the responses given by ChatGPT-3.5 in its two trials and separately compared the responses given by ChatGPT-4 in its two trials. The κ coefficient measures the agreement between these two sets of responses, providing an indication of the reliability of the AI’s performance across different attempts.

### Ethical Considerations

Members of the Dr. Balmis General University Hospital Ethics Review Board evaluated this project and stated that this committee was not competent to evaluate studies of this type, as they do not encompass human subjects, the use of biological samples, or personal data. Therefore, ethics committee approval was not required for the execution of this study.

## Results

### Neurologists’ Performance

In the examination under study, 120 neurologists participated. Their median score was 5.91 (IQR: 4.93-6.76) out of 10, with an SD of 1.40. The Kolmogorov-Smirnov test confirmed the normal distribution of these scores. Of these 120 neurologists, 32 did not pass the examination.

### ChatGPT-3.5 Performance

ChatGPT-3.5, acting as a hypothetical 121st participant, showed varying results in different attempts. In its first attempt, it answered 41 out of 77 questions correctly, and in another attempt, it managed 42 correct answers. ChatGPT-3.5’s scores were 3.77 and 3.94, respectively, in these attempts. However, it failed to reach the examination’s passing threshold. Specifically, it answered 32 out of 65 (49.2%) of the clinical neurology and 3.5 out of 12 (29.2%) of the health legislation questions incorrectly, leading to an overall error rate of 35.5 out of 77 (46.1%).

### ChatGPT-4 Performance

**Figure 1.**
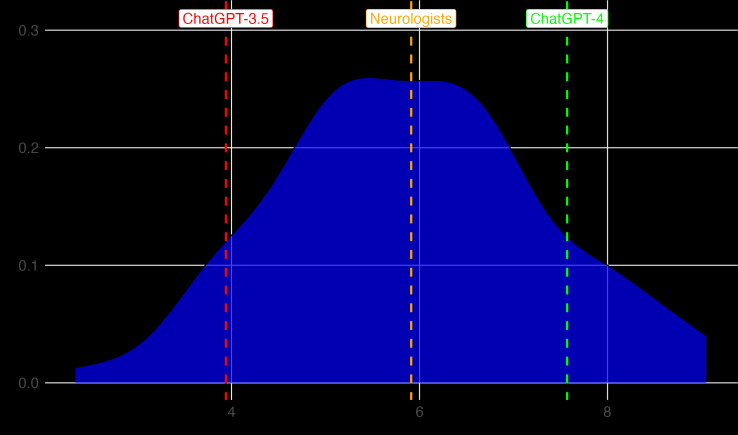
Distribution of neurologists’ examination scores. The graph shows the median performance of neurologists and the highest scores of ChatGPT-3.5 and ChatGPT-4 within the overall score distribution.

ChatGPT-4 demonstrated a more robust performance, correctly answering 62 and 63 out of 77 questions, respectively, in both the attempts, achieving a score of 7.57 out of 10 on its best attempt. This score would have qualified it to pass the examination, ranking it 17th out of the 122 candidates (which includes the 120 neurologists and both ChatGPT versions). ChatGPT-4’s error rate was 11.05 wrong answers out of 65 (17%) in clinical neurology questions and 3 out of 12 (25%) in legal questions. Figure 1 compares the score distribution of the neurologists who took the examination with the performances of ChatGPT-3.5 and ChatGPT-4.

### Concordance Analysis and Complexity-Based Performance

The κ coefficient for ChatGPT-3.5 was 0.686, measuring the consistency of its responses across attempts. ChatGPT-4’s κ coefficient was slightly higher at 0.725. Both models showed a high level of consistency in their performances across different attempts, with a mere 1.25% variation in their scores. Table 1 presents the performance data of each model and attempt, broken down by Bloom’s Taxonomy question classifications.

Based on Bloom’s Taxonomy, lower-order questions included tasks such as defining terms, recalling facts, and understanding basic concepts (eg, “Which lesion causes ideomotor apraxia?”). Higher-order questions required application, analysis, and evaluation (eg, “Given the following symptoms, what is the most likely diagnosis?”).

**Table 1.** Comparative performance analysis of ChatGPT-3.5 and ChatGPT-4 models on the examination: accuracy across attempts and question difficulty levels.

Model andattempt		Overall accuracy (%)	Accuracy on lower-order questions (%)	Accuracy on higher-order questions (%)
ChatGPT-3.5				
Attempt 1		53.25	54.84	52.17
Attempt 2		54.55	54.84	54.35
ChatGPT-4				
Attempt 1		81.82	77.42	84.78
Attempt 2		80.52	80.65	80.43

## Discussion

This study’s comparative analysis between ChatGPT and neurologists in a real medical examination offers valuable insights into the current abilities and limitations of AI in the assessment of medical knowledge. We selected ChatGPT, instead of other LLMs such as Gemini or Bard, for our study due to its well-documented performance in medical examinations, robust and user-friendly API facilitating easy integration and comprehensive testing, and its popularity and widespread usage, making it one of the most commonly used LLMs in the world as of December 2023.

ChatGPT has been able to pass the medical license examinations of several countries such as the United States [14], Germany [15], China [16], Japan [7], Saudi Arabia [17], Poland [18], and Spain [19]. Furthermore, ChatGPT has been able to pass the medical examination of a growing list of different medical specialties: anesthesiology [20], nuclear medicine [21], ophthalmology [22], otolaryngology [23], radiology [24], neurosurgery [25], and neurology [26,27].

A key strength of our study is its real-world setting—an actual competitive examination undertaken by 120 practicing neurologists, who were competing for specialized positions within the Valencian Health Service. This examination provides a tough and high-pressure assessment of their expertise, reflecting the pressures and complexities encountered in highly specialized and competitive scenarios. The range of scores among the neurologists serves as a human benchmark, highlighting the variability in medical expertise. This variability underlines the dynamic and individual nature of medical knowledge, and provides a realistic benchmark for assessing the capabilities of AI tools such as ChatGPT in professional scenarios. However, the focus on the Valencian Community might limit the generalizability of the findings to other regions or countries.

ChatGPT-3.5’s performance, though notable, reveals complexities. It accurately answered 42 (54.5%) of the questions in its best attempt, surpassing only 6 attending neurologists and failing to pass the examination. If ChatGPT-3.5 were a real examination participant, it would rank 116th out of 122 candidates—indicating room for improvement. The disparity in its performance between legal and neurology questions prompts further investigation into its decision-making processes. In contrast, ChatGPT-4’s performance shows significant improvement over ChatGPT-3.5. In the demanding neurology specialist examination, ChatGPT-4 not only surpassed its predecessor but also outperformed 103 of 120 human medical specialists. This marks a substantial advance in the model’s handling of specialized medical knowledge and suggests its potential as a tool in medical education and decision-making.

The study design we implemented did not include mechanisms for ChatGPT to explain or reason its answers, which limits our ability to evaluate the types of errors made by the AI models, such as differentiating between content errors and question interpretation errors. We did not prompt ChatGPT to provide explanations for its responses, and thus, we cannot perform a detailed analysis of its reasoning processes. This limitation highlights a gap in our study, as we were unable to analyze the types of errors made by ChatGPT. Future research should incorporate prompts for AI models to explain their answers, which would enable a deeper analysis of content errors versus question interpretation errors.

We calculated κ coefficients to assess the consistency of responses between trials for ChatGPT-3.5 and ChatGPT-4. The κ coefficient was 0.686 for ChatGPT-3.5 and 0.725 for ChatGPT-4, both indicating substantial but not perfect agreement. The slightly higher κ coefficient for ChatGPT-4 suggests improved reliability; however, the concordance is still not at a level that can be fully trusted without human oversight. This underscores the necessity for clinicians to critically evaluate AI responses and reasoning, reinforcing the principle that “two heads are better than one.” Future iterations should aim for even higher consistency, particularly in high-stakes fields such as neurology.

Unlike most existing literature that evaluates AI in English [28], our study probes ChatGPT’s performance in Spanish, a vital consideration for global medical applications given the variation in medical terminology and nuances across languages. The latest edition of the Cervantes Institute yearbook provides some data that reflect the magnitude of Spanish today [29]. It is the fourth most commonly used language globally and the third most widely used language on the internet. Two studies have analyzed the performance of ChatGPT versions 3.5 and 4 in the Spanish examination akin to the United States Medical Licensing Examination (USMLE) [19,30]. In the first study, ChatGPT-4 correctly answered 158 out of 182 (86.8%) of the questions, while in the second study, which focused solely on rheumatology questions, it correctly answered 134 out of 143 (93.7%) of the questions. In the first study, questions were prompted in both English and Spanish, with no significant differences observed. These data suggest that the performance of ChatGPT in Spanish in medical examinations is comparable to its performance in English.

ChatGPT sometimes provides confident answers that are meaningless when considered in the light of common knowledge in these areas. This phenomenon has been described as “artificial hallucination” [31]. This overconfidence was also observed in a neurology board-style examination [26] and in our study. Although the prompt for each question stated that “The objective is to achieve the maximum score. The score is equal to the number of correct answers minus incorrect answers divided by 3. So, if you are unsure about a question is better not to answer it in order to achieve the maximum possible score,” ChatGPT-3.5 and ChatGPT-4 answered all the questions. This behavior, known as “artificial hallucination,” poses serious risks in medical education, as overconfident yet wrong responses can mislead educators and students, potentially compromising patient safety and care quality. The AI’s inability to accurately gauge its confidence level and the appropriateness of not responding raises ethical concerns, especially in high-stakes environments such as neurology where precise knowledge and cautious decision-making are critical. To mitigate these risks, it is crucial to ensure that AI complements rather than replaces human judgment, with safeguards to prevent overreliance on AI. Training AI to recognize its limitations and abstain from responding when uncertain is essential to maintaining the integrity and safety of medical practice.

In contrast to another study where both models demonstrated weaker performance in tasks requiring higher-order thinking compared with questions requiring only lower-order thinking [26], our research revealed that ChatGPT’s performance remained consistent across tasks demanding both higher-order and lower-order thinking.

The ability of AI models, such as ChatGPT, to successfully pass medical examinations raises significant questions about the nature and effectiveness of these examinations. It is not just about what AI can do, but also what these examinations are really testing. This leads us to consider whether these exams accurately measure the real-world skills and knowledge essential for medical professionals. To address this, we propose several key areas of focus:

1. Uniquely human skills: More emphasis should be placed on assessing skills unique to human practitioners, such as clinical reasoning (gathering information, developing differential diagnosis, and justifying decision-making process), ethical judgment, and empathetic communication. These are vital yet challenging to quantify aspects of medicine, such as empathy, ethics, and patient-centered care. Developing methods to evaluate these skills could greatly benefit the medical field. Specifically, we propose the use of interactive patient simulations in which candidates must gather information directly from the patient. While current AI models can imitate specialist performance in clinical reasoning and developing differential diagnoses, the information provided to these models should be obtained through interactions with human specialists.
2. Application in real-world scenarios: Examinations should evolve to test the practical application of medical knowledge in real-life situations. This includes assessing abilities in diagnosis and treatment planning within complex clinical contexts, ensuring that professionals are prepared for real-world challenges. Additionally, allowing the use of LLM interfaces and other search engines during some examinations can simulate real-world conditions where clinicians have access to various technological aids. This approach not only tests their knowledge but also evaluates their critical thinking and ability to effectively search for and apply relevant information. Integrating these technologies into examinations can help improve clinicians’ performance by fostering skills that are essential in modern medical practice.
3. Interdisciplinary skills: Given the interdisciplinary nature of modern health care, examinations should also focus on teamwork, collaboration, and communication skills. They should assess the ability of medical professionals to integrate information across various specialties, reflecting the collaborative environment of contemporary health care.
4. Focus on continual learning: To motivate and teach lifelong learning, we need to shift our focus from merely teaching information retrieval to fostering skills in critical appraisal, problem-solving, and continuous professional development. While GPT can efficiently retrieve information, it is essential for medical professionals to critically appraise and apply this information. Future examinations should include components where candidates review and critique recent research articles, identifying strengths, weaknesses, and the applicability of findings to clinical practice. This ensures clinicians develop the ability to evaluate the quality and relevance of the information they encounter. Additionally, presenting candidates with novel clinical guidelines or emerging evidence in examinations will require them to integrate new information into their practice. This scenario-based assessment evaluates their ability to stay current with ongoing advancements and incorporate new knowledge effectively into clinical decision-making. Emphasizing self-directed learning and the use of various educational resources will help clinicians remain adaptable and proficient throughout their careers.

In summary, while AI passing medical examinations is an impressive feat, it highlights the need for evolution in medical education and assessment, ensuring that they measure the skills and knowledge that future medical professionals will truly need.

### Conclusion

Our study reveals the nuanced interplay between AI and human expertise in neurology, highlighting ChatGPT’s potential as a medical knowledge resource. Despite its promising performance, the variability in both AI and human responses calls for a careful, measured integration of AI into medical practice.

The combination of AI and human expertise could significantly enhance medical education and practice. However, this integration must prioritize patient care and safety, ensuring that AI complements rather than replaces human judgment.

In summary, this research contributes to the ongoing narrative of AI in health care and sets the stage for further exploration into refining AI for specialized medical uses. The focus remains on harnessing AI to support, not supplant, the invaluable insights of medical professionals.

## Supplementary material

10.2196/56762 Multimedia Appendix 1 Initial prompt for each question and scores of the 120 participating neurologists.

## Abbreviations

AI: artificial intelligence
API: application programming interfaces
LLM: large language model
USMLE: United States medical licensing examination
