# Supplementary material for: Evaluating AI Competence in Specialized Medicine: Comparative Analysis of ChatGPT and Neurologists in a Neurology Specialist Examination in Spain
Source: JMIR Med Educ. 2024 Nov 14;10:e56762. doi: 10.2196/56762 (PMC11611784; doi:10.2196/56762)
Supplement: Multimedia Appendix 1 [file mededu-v10-e56762-s001.docx]

**SUPPLEMENT 1.**

**Prompt for each question**

The prompt used to analyze each question was (in Spanish): *Me gustaría probar tu habilidad para realizar un examen sobre neurología clínica y temas de legislación general y sanitaria. Las preguntas están en español y tienen 4 opciones, de las cuales solo una es correcta. El objetivo es alcanzar la máxima puntuación. La puntuación es igual al número de respuestas correctas menos las respuestas incorrectas divididas por tres. Por lo tanto, si no estás seguro sobre una pregunta, es mejor no responderla para lograr la máxima puntuación posible.*

Translation: I would like to test your ability to do a test about clinical neurology and general public and health legislation topics. The questions are in Spanish and have 4 options, only one is correct. The objective is to achieve the maximum score. The score is equal to the number of correct answers minus incorrect answers divided by three. So, it you are unsure about a question is better not to answer it in order to achieve the maximum possible score.

**Table S1.** Participant Scores Ordered from Highest to Lowest, Including the Results of ChatGPT-3.5 and ChatGPT-4

| **Position** | **Score** | **Participant Type** |
| --- | --- | --- |
| 1 | 9.05 | Neurologist |
| 2 | 8.96 | Neurologist |
| 3 | 8.96 | Neurologist |
| 4 | 8.79 | Neurologist |
| 5 | 8.49 | Neurologist |
| 6 | 8.4 | Neurologist |
| 7 | 8.36 | Neurologist |
| 8 | 8.18 | Neurologist |
| 9 | 8.01 | Neurologist |
| 10 | 7.97 | Neurologist |
| 11 | 7.97 | Neurologist |
| 12 | 7.92 | Neurologist |
| 13 | 7.88 | Neurologist |
| 14 | 7.88 | Neurologist |
| 15 | 7.79 | Neurologist |
| 16 | 7.75 | Neurologist |
| **17** | **7.57** | **ChatGPT4** |
| 18 | 7.49 | Neurologist |
| 19 | 7.4 | Neurologist |
| 20 | 7.36 | Neurologist |
| 21 | 7.23 | Neurologist |
| 22 | 7.23 | Neurologist |
| 23 | 7.1 | Neurologist |
| 24 | 7.06 | Neurologist |
| 25 | 7.06 | Neurologist |
| 26 | 6.97 | Neurologist |
| 27 | 6.93 | Neurologist |
| 28 | 6.88 | Neurologist |
| 29 | 6.8 | Neurologist |
| 30 | 6.8 | Neurologist |
| 31 | 6.8 | Neurologist |
| 32 | 6.75 | Neurologist |
| 33 | 6.75 | Neurologist |
| 34 | 6.71 | Neurologist |
| 35 | 6.71 | Neurologist |
| 36 | 6.67 | Neurologist |
| 37 | 6.67 | Neurologist |
| 38 | 6.67 | Neurologist |
| 39 | 6.67 | Neurologist |
| 40 | 6.58 | Neurologist |
| 41 | 6.58 | Neurologist |
| 42 | 6.54 | Neurologist |
| 43 | 6.49 | Neurologist |
| 44 | 6.45 | Neurologist |
| 45 | 6.41 | Neurologist |
| 46 | 6.36 | Neurologist |
| 47 | 6.32 | Neurologist |
| 48 | 6.32 | Neurologist |
| 49 | 6.28 | Neurologist |
| 50 | 6.23 | Neurologist |
| 51 | 6.23 | Neurologist |
| 52 | 6.19 | Neurologist |
| 53 | 6.19 | Neurologist |
| 54 | 6.15 | Neurologist |
| 55 | 6.1 | Neurologist |
| 56 | 6.1 | Neurologist |
| 57 | 6.06 | Neurologist |
| 58 | 5.97 | Neurologist |
| 59 | 5.89 | Neurologist |
| 60 | 5.89 | Neurologist |
| 61 | 5.85 | Neurologist |
| 62 | 5.85 | Neurologist |
| 63 | 5.85 | Neurologist |
| 64 | 5.85 | Neurologist |
| 65 | 5.8 | Neurologist |
| 66 | 5.8 | Neurologist |
| 67 | 5.76 | Neurologist |
| 68 | 5.63 | Neurologist |
| 69 | 5.63 | Neurologist |
| 70 | 5.59 | Neurologist |
| 71 | 5.5 | Neurologist |
| 72 | 5.5 | Neurologist |
| 73 | 5.41 | Neurologist |
| 74 | 5.37 | Neurologist |
| 75 | 5.37 | Neurologist |
| 76 | 5.37 | Neurologist |
| 77 | 5.37 | Neurologist |
| 78 | 5.37 | Neurologist |
| 79 | 5.28 | Neurologist |
| 80 | 5.24 | Neurologist |
| 81 | 5.2 | Neurologist |
| 82 | 5.2 | Neurologist |
| 83 | 5.2 | Neurologist |
| 84 | 5.2 | Neurologist |
| 85 | 5.15 | Neurologist |
| 86 | 5.11 | Neurologist |
| 87 | 5.11 | Neurologist |
| 88 | 5.07 | Neurologist |
| 89 | 5.07 | Neurologist |
| 90 | 4.98 | Neurologist |
| 91 | 4.94 | Neurologist |
| 92 | 4.89 | Neurologist |
| 93 | 4.89 | Neurologist |
| 94 | 4.89 | Neurologist |
| 95 | 4.85 | Neurologist |
| 96 | 4.81 | Neurologist |
| 97 | 4.81 | Neurologist |
| 98 | 4.76 | Neurologist |
| 99 | 4.76 | Neurologist |
| 100 | 4.72 | Neurologist |
| 101 | 4.68 | Neurologist |
| 102 | 4.63 | Neurologist |
| 103 | 4.59 | Neurologist |
| 104 | 4.37 | Neurologist |
| 105 | 4.33 | Neurologist |
| 106 | 4.24 | Neurologist |
| 107 | 4.2 | Neurologist |
| 108 | 4.16 | Neurologist |
| 109 | 4.11 | Neurologist |
| 110 | 3.98 | Neurologist |
| 111 | 3.94 | Neurologist |
| 112 | 3.94 | Neurologist |
| 113 | 3.81 | Neurologist |
| 114 | 3.77 | Neurologist |
| 115 | 3.77 | Neurologist |
| **116** | **3.77** | **ChatGPT3.5** |
| 117 | 3.64 | Neurologist |
| 118 | 3.59 | Neurologist |
| 119 | 3.55 | Neurologist |
| 120 | 3.55 | Neurologist |
| 121 | 2.77 | Neurologist |
| 122 | 2.34 | Neurologist |
